# Association between waist circumference and fatty liver disease in older adult population: a cross-sectional study in Urumqi

**DOI:** 10.3389/fpubh.2025.1620261

**Published:** 2025-07-08

**Authors:** Mingdong Zhang, E. Zhao, Gaofeng Sun

**Affiliations:** ^1^School of Public Health, Xinjiang Medical University, Urumqi, China; ^2^Department of Chronic Non-Communicable Disease Prevention and Control, Urumqi Center for Disease Control and Prevention, Urumqi Municipal Health Supervision Institute, Urumqi, China

**Keywords:** older adult population, waist circumference, fatty liver, machine learning, restricted cubic spline

## Abstract

**Objective:**

This study aims to explore the correlation between waist circumference and the prevalence of fatty liver disease in the older adult population in Urumqi.

**Methods:**

Through cluster random sampling of healthcare institutions within the urban districts of Urumqi, a final cohort of 3,907 participants was enrolled from three institutions. In addition, the informed consent forms of the participants were obtained. Chi-square tests were used for univariate analysis between groups, and the data were divided into a training set and a test set in a 7:3 ratio. Variables were further screened using machine learning models such as random forest classifier and Lasso. Logistic regression and restricted cubic spline models were used to analyze the correlation between waist circumference and fatty liver disease.

**Results:**

The prevalence of fatty liver disease was 32.56%, with 31.54% in men and 33.40% in women. Multivariate logistic regression analysis showed that compared with non-central obesity, the risk of fatty liver disease in central obesity was significantly higher (*OR* = 1.768, 95% *CI*: 1.481–2.112). The restricted cubic spline model analysis showed that the risk of fatty liver disease increased with waist circumference in the older adult population. In the total population and the male group, waist circumference and central obesity showed a nonlinear relationship, while in the female group, those below 75 years old, and those 75 and older, a linear relationship was observed.

**Conclusion:**

Controlling waist circumference is important for the prevention of fatty liver disease. The older adult population in Urumqi should pay attention to the risks posed by increasing waist circumference.

## 1 Introduction

By 2022, the prevalence of fatty liver disease among adults in China reached 44.39% (1), making fatty liver the leading liver disease in China. Fatty liver not only is the main cause of liver cirrhosis and hepatocellular carcinoma (2), but also increases the risk of cardiovascular disease, type 2 diabetes, chronic kidney disease, and certain cancers (3). The older adult population has a higher risk of developing fatty liver disease due to factors such as aging, reduced physical activity, and changes in metabolism (4–6). As a high-risk group for fatty liver disease, the prevention and treatment of fatty liver in the older adult population cannot be overlooked. Obesity, unhealthy dietary patterns, and metabolic disorders are considered primary risk factors for fatty liver disease (7), and BMI and metabolism-related indicators are also major predictive factors for fatty liver disease prevalence (8). Current core prevention and management strategies for fatty liver disease focus on lifestyle interventions. These include improving metabolic disorders through dietary adjustments, increased physical activity, and weight loss (9). Currently, there are no long-term approved pharmacotherapy specifically for fatty liver disease itself, with existing drugs primarily used for treating advanced disease or managing related comorbidities (10).

Waist circumference is currently the main indicator for diagnosing central obesity (11). Studies have shown that the prevalence of fatty liver disease in patients with central obesity is higher than in normal individuals (12), and compared to BMI, waist circumference is more effective in identifying and predicting visceral fat obesity and related diseases (13, 14). Therefore, waist circumference is of significant importance in predicting the risk of fatty liver disease, but there are relatively few studies on the correlation between waist circumference and the risk of fatty liver disease. This study aims to use the restricted cubic spline model to analyze the association between waist circumference and the prevalence of fatty liver disease in the older adult population, providing a scientific basis for the prevention and treatment of fatty liver disease in this group.

## 2 Materials and methods

### 2.1 Study subjects

**Figure 1:**
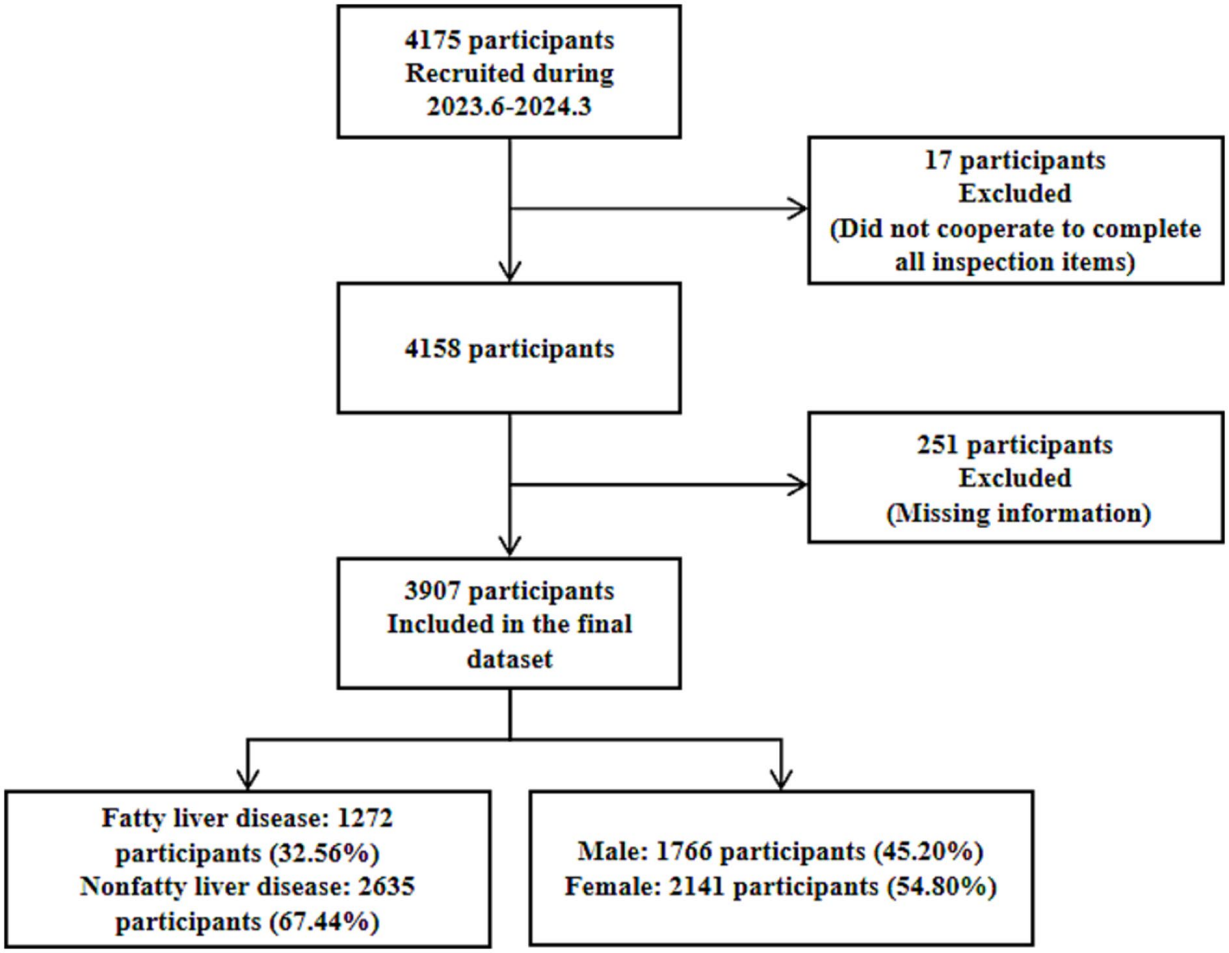
Flowchart of selection for participants.

This study was conducted using cluster random sampling of medical institutions within the urban area of Urumqi City. The participants were older adult individuals aged 65 years or older who underwent health examinations at three medical institutions within the urban area of Urumqi City between June 2023 and March 2024. Inclusion criteria: (1) Having resided in the urban area of Urumqi City for at least 6 months. (2) Aged 65 years or older. (3) Able to independently cooperate to complete the survey. Exclusion criteria: (1) Eligible individuals unwilling to participate in the study. (2) Individuals with psychiatric disorders or end-stage diseases. A total of 4,175 participants were initially enrolled. Seventeen participants who failed to complete all required examinations were excluded, and 251 participants with missing information (missing basic information, health status, or disease history) were excluded. Finally, 3,907 participants were included in the study. Among these, there were 1,766 males and 2,141 females. The flow chart is presented in Figure 1. This study was reviewed and approved by the Ethics Committee of the Urumqi Center for Disease Control and Prevention, with an ethics review approval number of 2024-C111.

### 2.2 Data collection and measurement

Data were obtained through face-to-face questionnaires, laboratory tests, and physical examinations. All data collection and measurements were performed by experienced medical personnel who underwent standardized uniform training. The questionnaire covered basic demographic characteristics, lifestyle habits (such as smoking, drinking, exercise frequency, and dietary habits), laboratory tests (including fasting blood glucose, total cholesterol, triglycerides, high-density lipoprotein cholesterol, low-density lipoprotein cholesterol, etc.). Laboratory tests were performed using a fully automated biochemical analyzer (Mindray BS-600M, Shenzhen, China). Physical examination items included height, weight, waist circumference, blood pressure, electrocardiogram (ECG), abdominal ultrasound, etc. Height was measured using an electronic stadiometer (CAMRY iHT721D, Zhongshan City, China). Weight was measured using an electronic weighing scale (CAMRY EF881H, Zhongshan City, China). Waist circumference was measured at the midpoint between the iliac crest and the lower rib margin using an electronic digital soft tape (SENSSUN iS620B, Zhongshan City, China), to the nearest 0.1 cm. The mean of two measurements was calculated. Blood pressure was measured using an electronic sphygmomanometer (OMRON HEM-7111, Kyoto, Japan). Systolic blood pressure (SBP) and diastolic blood pressure (DBP) were measured three times, and the average of the three readings was calculated. Electrocardiogram (ECG) was performed using a fully digital multi-channel ECG machine (Mindray BeneHeart R700, Shenzhen, China). Participants were placed in the supine position in a quiet environment during resting conditions, and a continuous recording for 10 s was obtained. Ultrasound examinations (liver, cardiovascular) were performed using a color Doppler ultrasound system (Mindray DC-80, Shenzhen, China) with its matching convex array probe. All equipment used in the study met the Chinese national standards.

### 2.3 Definition of fatty liver disease

Fatty liver disease definition: Based on abdominal ultrasound features including liver echogenicity, deep attenuation, and clarity of ductal structures, fatty liver was diagnosed according to the Chinese Expert Recommendations for the Standardized Diagnosis and Treatment of Fatty Liver Diseases (2019 Revision) criteria. Mild: Enhanced near-field echoes in the liver parenchyma (higher than kidney/spleen), deep-field echo attenuation ≤1/3, and clear intrahepatic ductal structures. Moderate: Deep-field echo attenuation between 1/3 and 2/3, and blurred ductal structures. Severe: Deep-field echo attenuation >2/3, non-visible ductal structures, accompanied by hepatomegaly and rounded edges (15). Fatty liver was diagnosed upon meeting any of the criteria for mild, moderate, or severe. Central obesity definition: Waist circumference was measured using an electronic digital soft tape at the midpoint between the iliac crest and the lower rib margin. Based on the Chinese Guidelines for the Diagnosis and Treatment of Obesity (2024 Edition), central obesity was defined as a mean waist circumference (from two measurements) ≥ 90 cm for males or ≥85 cm for females. BMI definition: BMI < 18.5 kg/m^2^ was classified as underweight, 18.5 kg/m^2^ ≤ BMI < 24 kg/m^2^ as normal weight, 24 kg/m^2^ ≤ BMI < 28 kg/m^2^ as overweight, and BMI ≥ 28 kg/m^2^ as obese (16). Hypertension definition: Defined as systolic blood pressure (SBP) ≥ 140 mmHg and/or diastolic blood pressure (DBP) ≥ 90 mmHg, a previous medical history of hypertension, or current antihypertensive medication use (17). Diabetes definition: Defined as fasting plasma glucose ≥7.0 mmol/L, previously diagnosed with diabetes, or using glucose-lowering medications (18). Dyslipidemia definition: Defined upon meeting any one of the following criteria: total cholesterol ≥6.22 mmol/L, triglycerides ≥2.26 mmol/L, low-density lipoprotein cholesterol ≥4.14 mmol/L, or high-density lipoprotein cholesterol <1.04 mmol/L (19). Electrocardiogram (ECG) definition: Based on ECG findings, an abnormal ECG was defined as the presence of any of the following: premature beats, arrhythmias, ST-segment changes, T-wave changes, or abnormal Q waves. Aortic sclerosis definition: Based on cardiovascular ultrasound findings, aortic sclerosis was defined as the presence of any one of the following: aortic wall thickening, increased echogenicity, detection of plaque (soft plaque, hard plaque, or mixed plaque), or luminal narrowing.

### 2.4 Statistical methods

SPSS 26.0 statistical software was used, and data were presented as mean ± standard deviation, and composition ratio (%), with chi-square tests used for analysis between groups with different characteristics. Using R4.4.0 software, and leveraging the commonly used caret package, the original dataset was stratified by the target variable and partitioned into a training set (70%) and a test set (30%). The random forest classifier with default parameters (n_estimators = 100) and the Lasso model with default parameters (alpha = 1.0) were selected. The random forest classifier was implemented using the Boruta package, while the Lasso model was implemented using the caret package. Models were built on both the training set and the test set. The area under the receiver operating characteristic curve (AUC) scores were compared. This step is based on the pROC package (AUC confidence intervals are calculated by the bootstrap algorithm of the package). The intersection of significant features identified by both models (variables deemed important by both models) was used for feature selection. Following correlation analysis, these features were incorporated into the subsequent logistic regression model and restricted cubic spline (RCS) model. Categorical variables were encoded using ordinal encoding. Using R software version 4.4.0, adjusted logistic regression analyses were performed, incorporating different sets of factors. Based on the rms package within the software, and utilizing the Akaike Information Criterion (AIC) to select the knots for the restricted cubic spline (RCS) model, a dose–response relationship curve between waist circumference and fatty liver disease risk was established. The linearity assumption of the RCS model was tested using the likelihood ratio test.

## 3 Results

### 3.1 Basic information

A total of 3,907 older adult individuals were included in this study, with a fatty liver disease prevalence of 32.56%, and an average age of 72.23 ± 5.94 years. Among them, there were 1,766 males and 2,141 females, with the prevalence of fatty liver disease being 31.54% in males and 33.40% in females. A univariate chi-square test was conducted between different groups of older adult individuals, and the results showed that differences in age, BMI classification, central obesity, hypertension, diabetes, dyslipidemia, smoking status, drinking frequency, electrocardiogram (ECG), and aortic sclerosis were statistically significant (*p* < 0.05). Differences in gender, exercise frequency, and dietary habits were not statistically significant (*p* > 0.05), as shown in Supplementary Table 1.

### 3.2 Random forest classifier

**Figure 2:**
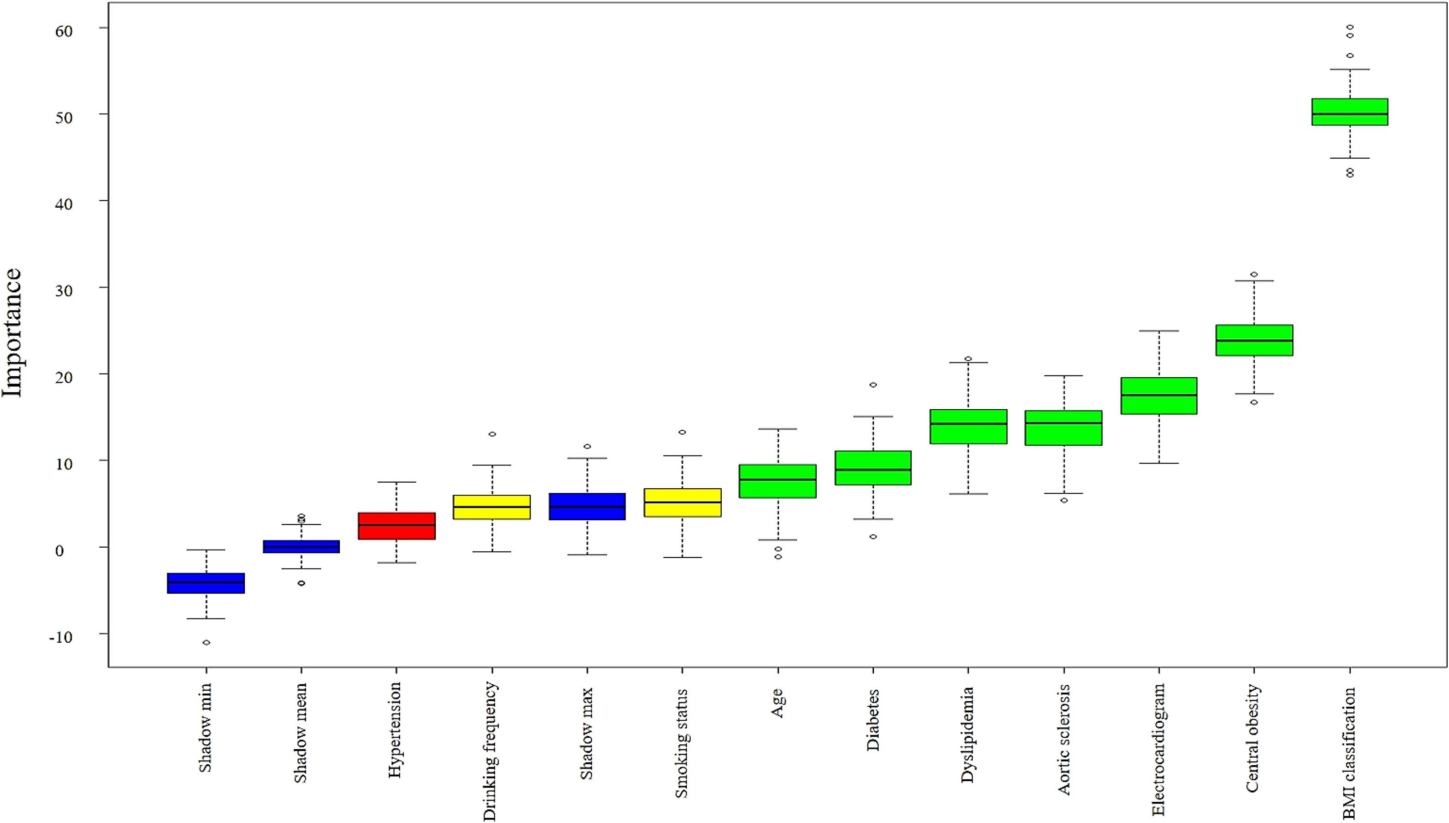
Boxplot of importance ranking for factors associated with fatty liver disease. Important features are shown in green boxes (7), tentative features in yellow boxes (2), and rejected features in red boxes (1). Shadow variables are displayed in blue boxes (3): minimum shadow value (Shadow min), maximum shadow value (Shadow max), mean shadow value (Shadow mean). The yellow box (Drinking frequency) contains discrete high-value points exceeding Shadow max, while the red box (Hypertension) contains values above Shadow min. These reflect random associations occurring in a small number of iterations. Following Boruta’s stability principle and the majority voting results, both features were still classified as tentative and rejected, respectively.

Whether having fatty liver disease was used as the dependent variable. Variables showing statistically significant differences in univariate chi-square tests were incorporated into the random forest classifier model established using the Boruta algorithm. This algorithm constructs “shadow variables” (blue) as randomized reference benchmarks. For the majority of the 99 iterations (convergence number of feature selection rounds) in the random forest classifier, variables were confirmed as important features (green) with significant influence on fatty liver disease when their importance exceeded the maximum shadow variable importance. Variables with importance between the minimum and maximum shadow variable importance were considered tentative features (yellow) with potential influence on fatty liver disease. Variables with importance below the minimum shadow variable importance were regarded as rejected features (red), indicating either no impact or collinearity issues regarding fatty liver disease. Ranked from highest to lowest importance, the results identified seven variables as important features (green): BMI classification, central obesity, electrocardiogram, aortic sclerosis, dyslipidemia, diabetes, and age, Two variables: smoking status and drinking frequency were identified as uncertain features (yellow). Hypertension was identified as one rejected feature (red). The importance ranking is shown in Figure 2.

### 3.3 LASSO model

**Figure 3:**
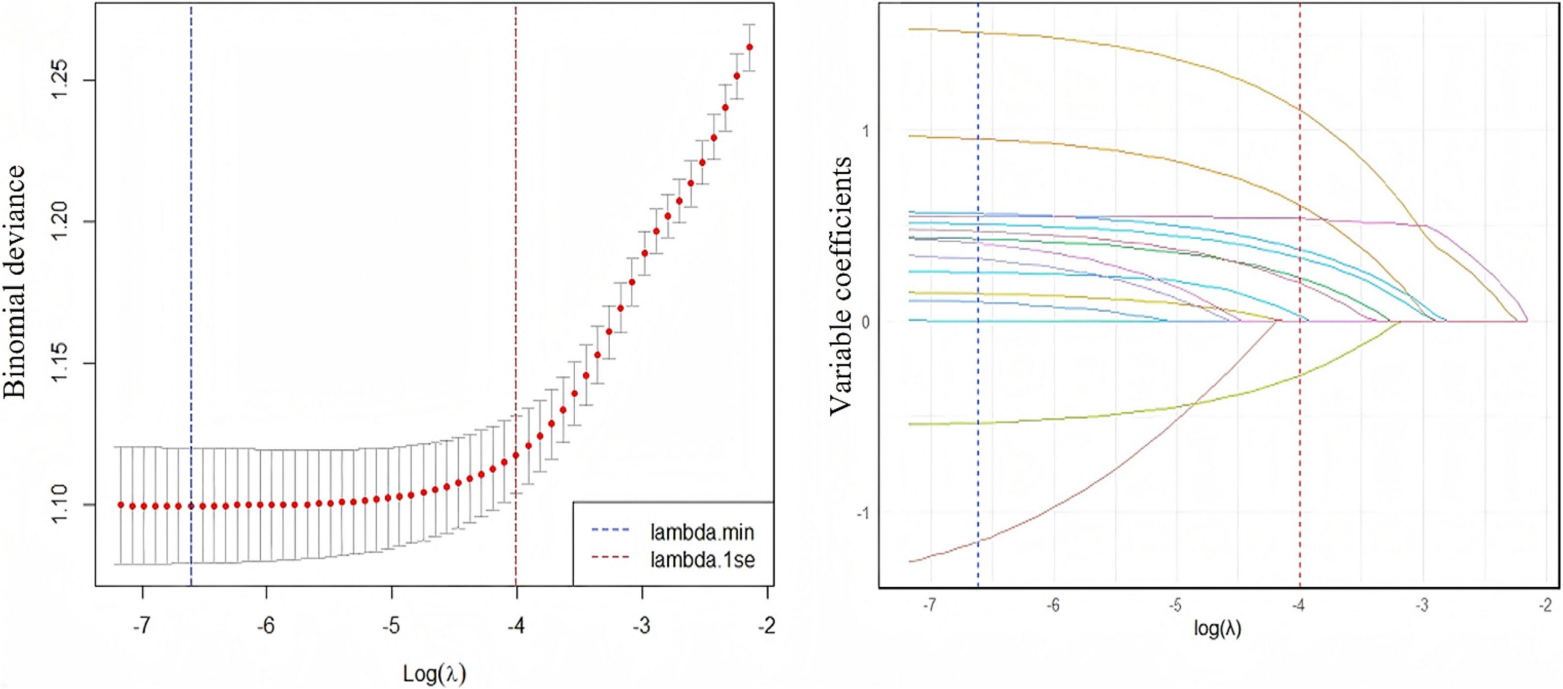
The relationship between log(*λ*) value and lasso regression coefficients (left), and the trend of variable coefficients corresponding to log(λ) value (right). Left panel: As log(*λ*) decreases, the mean binomial deviance (red line) declines rapidly before stabilizing. Minimal deviance is achieved at lambda.min (vertical line), indicating optimal predictive performance at this penalty strength. Right panel: Coefficients converge toward zero as log(*λ*) increases. At lambda.min, nine variables exhibit non-zero coefficients persistently distinct from zero, while all others are compressed to zero.

LASSO model was employed to identify predictors significantly associated with fatty liver disease from variables exhibiting statistical significance in univariate chi-square tests. To determine the optimal regularization strength, k-fold cross-validation (*k* = 5) was performed to compute the binomial deviance across varying *λ* values. The validation process partitioned the training set into 5 subsets. After iterative rotation and validation, the mean deviance was calculated, identifying λ yielding minimal deviance (lambda.min = 0.001344464), corresponding to log(λ) = −6.61176. The LASSO algorithm shrinks irrelevant variable coefficients exactly to zero, while retaining predictors with non-zero coefficients. This indicates that the contribution strength of these retained variables to predicting fatty liver disease is sufficient to overcome the regularization penalty. Leveraging this mechanism, nine key features with non-zero coefficients were selected: BMI classification, age classification, central obesity, drinking frequency, diabetes, electrocardiogram, dyslipidemia, aortic sclerosis, and smoking status, as visualized in Figure 3. This outcome reduced model dimensionality and mitigated overfitting risks. The model constructed using the cross-validation-selected lambda.min provides a reliable feature subset for subsequent analyses.

### 3.4 Model performance and feature selection

**Figure 4:**
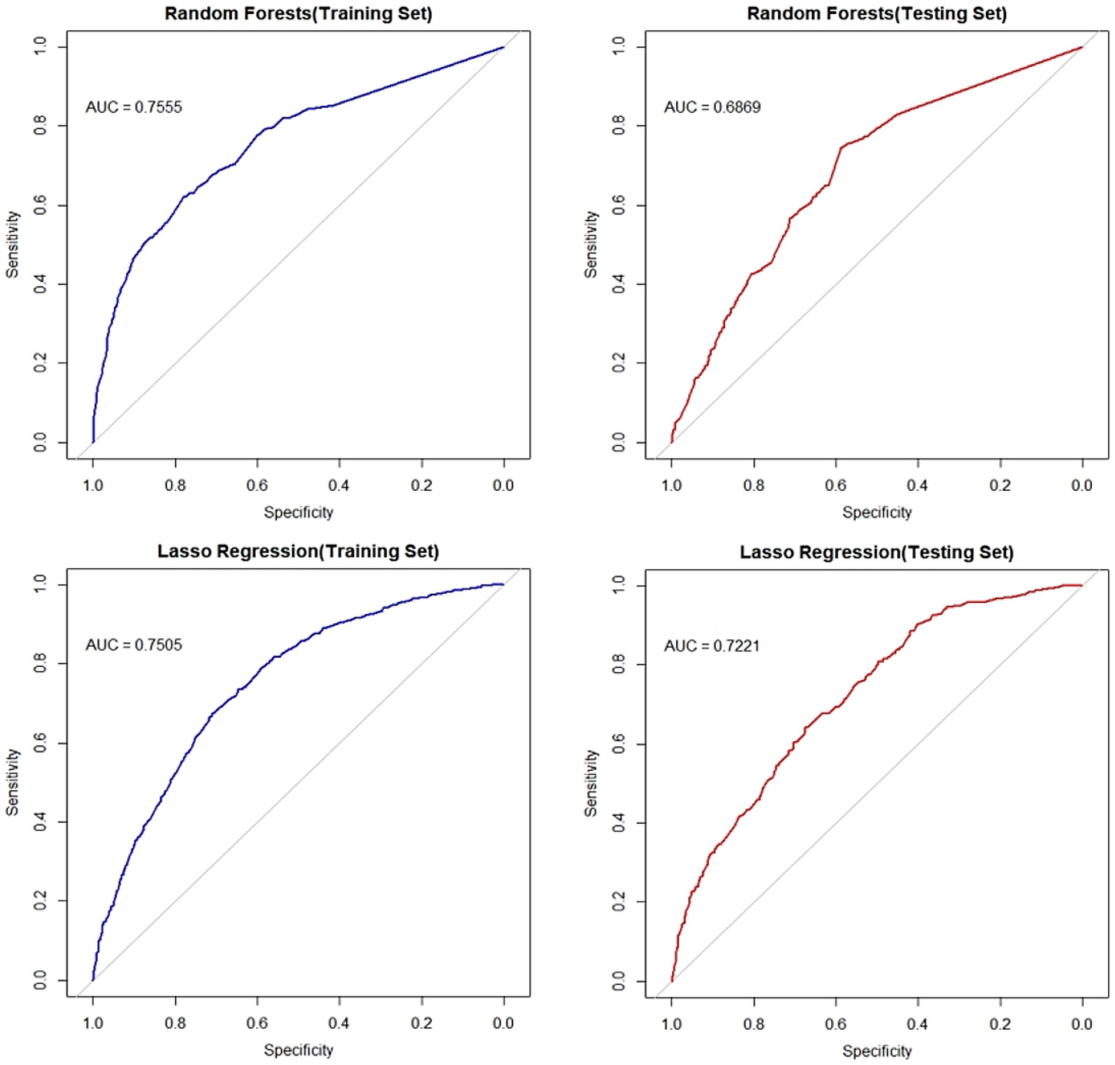
ROC curves of the random forest model and lasso regression model. The left panel displays ROC curves for both models on the training set, while the right panel shows curves for the test set. Blue lines indicate training set performance; red lines denote test set performance. Plots have 1-specificity on the *x*-axis and sensitivity on the *y*-axis.

The ROC curves of the random forest classifier and the LASSO model for both the training and testing sets were above the ROC random guessing line, indicating that both models performed better than random guessing. The AUC value of the random forest model for the training set was 0.7555 (95% *CI*: 0.7356–0.7754), and the AUC value for the testing set was 0.6869 (95% *CI*: 0.6552–0.7185). The AUC value of the LASSO regression model for the training set was 0.7505 (95% *CI*: 0.7316–0.7695), and the AUC value for the testing set was 0.7221 (95% *CI*: 0.6921–0.7521). The AUC values of both models for the training set were greater than 0.7, indicating that both models have good predictive performance for the risk of fatty liver disease in the older adult population. The intersection of the screening results of the two models was used as a variable to be included in the subsequent model. The ROC curves of the two models are shown in Figure 4.

### 3.5 Factor correlation analysis

**Figure 5:**
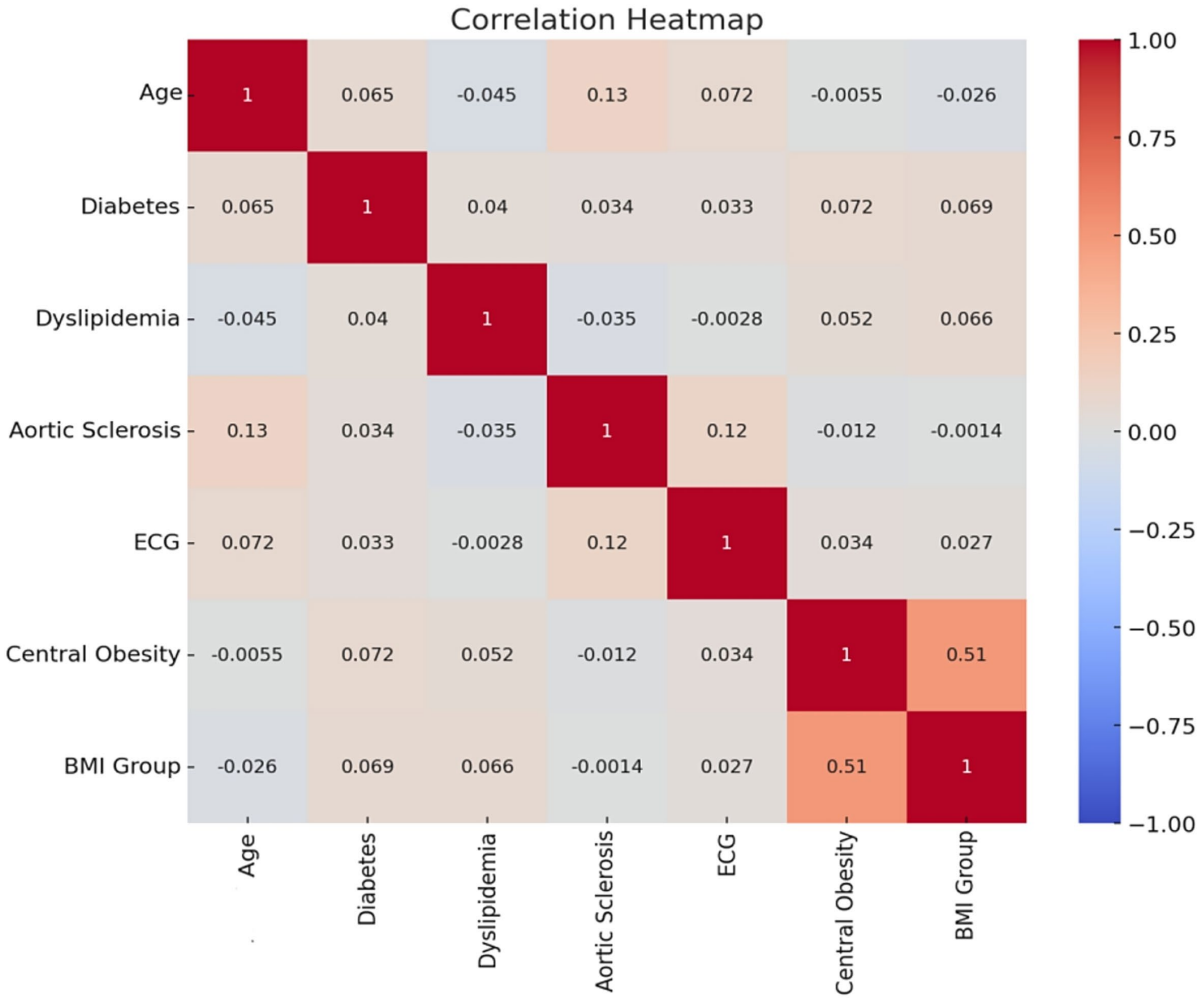
Factor correlations.

Correlation analysis was performed on the factors selected by the random forest classifier and lasso regression model, and no strong correlations were found between BMI classification, central obesity, electrocardiogram, aortic sclerosis, dyslipidemia, diabetes, and age classification. There was a positive correlation between BMI classification and central obesity with a correlation coefficient of 0.51, which did not reach the strong correlation threshold of 0.7, so no adjustments were made for these two factors. The correlation is shown in Figure 5.

### 3.6 Logistic regression analysis of central obesity and risk of fatty liver disease

The diagnostic criterion for central obesity is defined by waist circumference, so it is treated as a binary variable based on waist circumference. Therefore, a logistic regression model adjusting for different factors was used to explore the relationship between central obesity and fatty liver disease. BMI classification, central obesity, electrocardiogram, aortic sclerosis, dyslipidemia, diabetes, and age classification, which were selected by the random forest model from the training set data, were used as independent variables, and the presence of fatty liver disease (0 = no, 1 = yes) was used as the dependent variable to establish a multivariate logistic regression model. The results of univariate logistic regression in Model 1 showed that central obesity was significantly associated with fatty liver disease in the overall population, different gender subgroups (male group and female group), and different age subgroups (under 75 years old and 75 years and older) (*p* < 0.05). In Model 2, after adjusting for central obesity, age, and BMI classification, central obesity remained significantly associated with fatty liver disease in the overall population and different subgroups (*p* < 0.05). In Model 3, after further adjusting for central obesity, age, BMI classification, dyslipidemia, diabetes, electrocardiogram, and aortic sclerosis, central obesity remained significantly associated with fatty liver disease in both the overall population and different subgroups (*p* < 0.05), as shown in Table 1.

**Table 1:** Logistic regression analysis of central obesity and risk of fatty liver disease.

Group		Model 1		Model 2		Model 3	
		*OR* Value (95% *CI*)	*p* Value	*OR* Value (95% *CI*)	*p* Value	*OR* Value (95% *CI*)	*p* Value
Overall	Central obesity (Ref = No)	Ref (1.00)		Ref (1.00)		Ref (1.00)	
	Yes	3.239 (2.787–3.772)	<0.01	1.797 (1.511–2.138)	<0.01	1.768 (1.481–2.112)	<0.01
Male	Central obesity (Ref = No)	Ref (1.00)		Ref (1.00)		Ref (1.00)	
	Yes	3.846 (3.060–4.862)	<0.01	2.153 (1.659–2.804)	<0.01	2.117 (1.623–2.770)	<0.01
Female	Central obesity (Ref = No)	Ref (1.00)		Ref (1.00)		Ref (1.00)	
	Yes	2.822 (2.313–3.455)	<0.01	1.537 (1.218–1.942)	<0.01	1.525 (1.201–1.938)	<0.01
Under 75 years old	Central obesity (Ref = No)	Ref (1.00)		Ref (1.00)		Ref (1.00)	
	Yes	3.231 (2.711–3.86)	<0.01	1.842 (1.507–2.254)	<0.01	1.819 (1.483–2.235)	<0.01
75 years and older	Central obesity (Ref = No)	Ref (1.00)		Ref (1.00)		Ref (1.00)	
	Yes	3.356 (2.502–4.551)	<0.01	1.668 (1.186–2.360)	<0.01	1.606 (1.130–2.294)	0.01

Model 1: Univariate logistic regression analysis.

Model 2: Adjusted for age, and BMI classification.

Model 3: Adjusted for age, BMI classification, dyslipidemia, diabetes, electrocardiogram, and aortic sclerosis.

### 3.7 Correlation between waist circumference and fatty liver disease

Waist circumference was used as the independent variable, and fatty liver disease was used as the dependent variable to establish a restricted cubic spline model using Akaike information criterion (AIC). The AIC values of restricted cubic spline models with different knot points were compared. In general, 3–7 knots are considered appropriate for restricted cubic spline models (20). In this study, when 3 knots were selected, the AIC values for the overall population model, the gender subgroup model, and the age subgroup model were the smallest (AIC _overall_ = 4,337.857, AIC _male_ = 1,910.379, AIC _female_ = 2,419.118, AIC _under 75 years_ = 3,102.407, and AIC _75 years and older_ = 1,202.374). Based on the importance ranking from the random forest model using the training set, six factors (age classification, BMI classification, diabetes, dyslipidemia, electrocardiogram, and aortic sclerosis) were included in the restricted cubic spline model.

**Figure 6:**
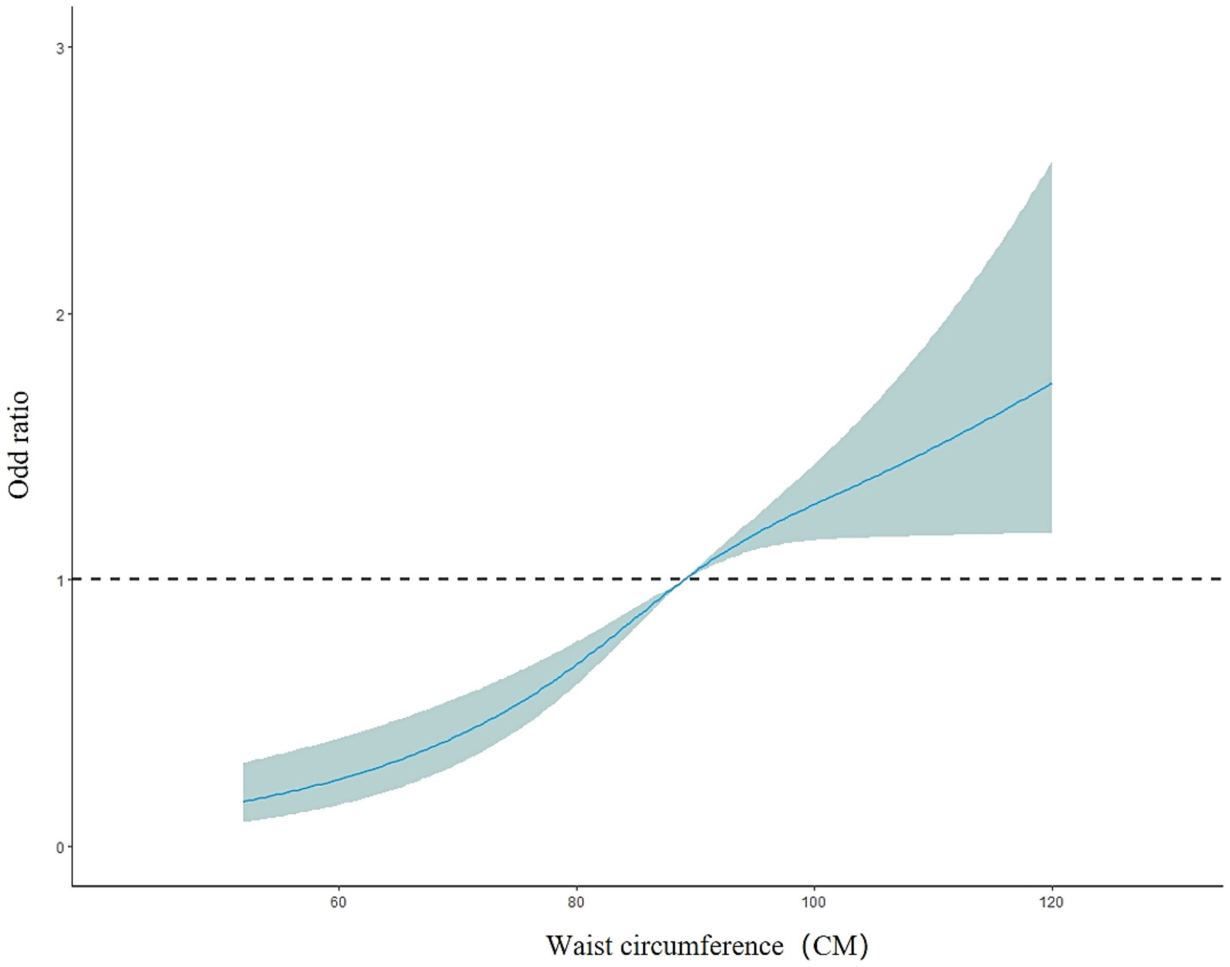
Dose–response relationship between waist circumference and the risk of fatty liver disease in the older adult population.

**Figure 7:**
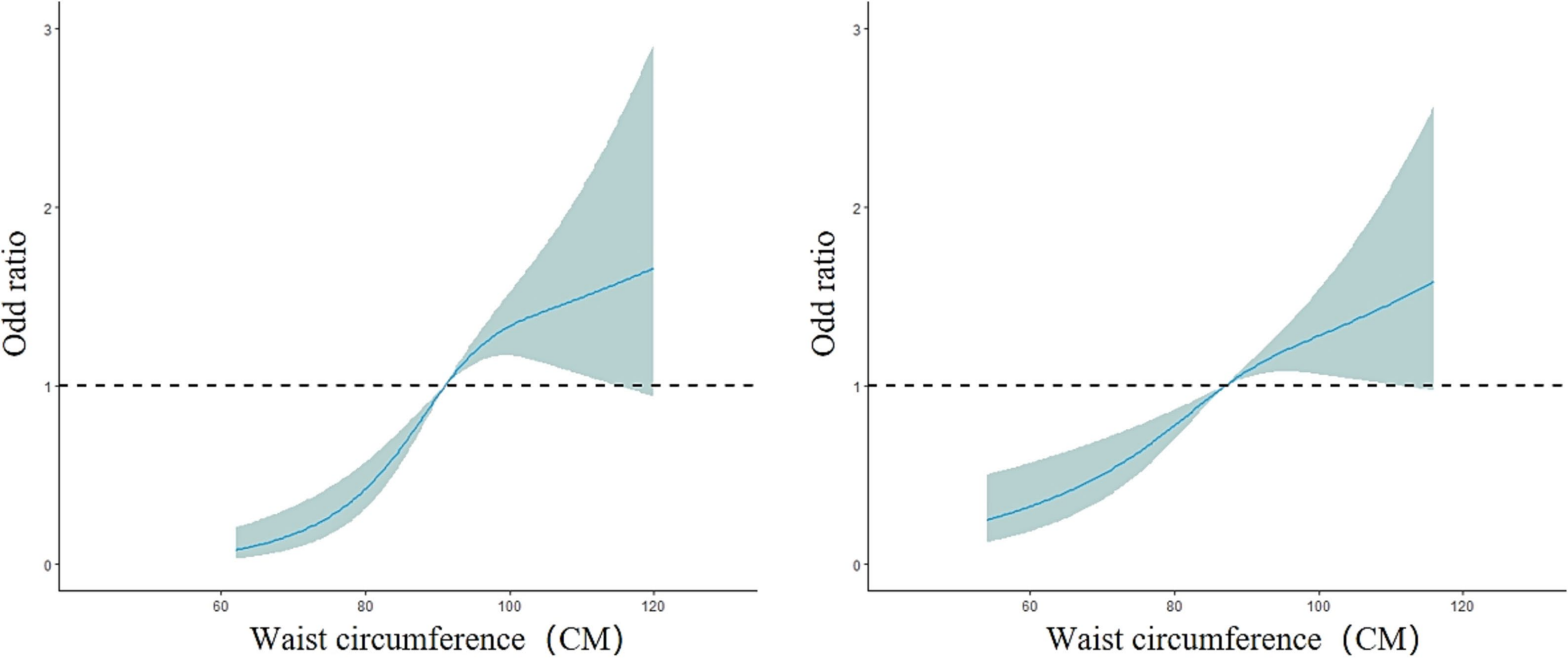
Dose–response relationship between waist circumference and the risk of fatty liver disease in the male group (left) and female group (right).

**Figure 8:**
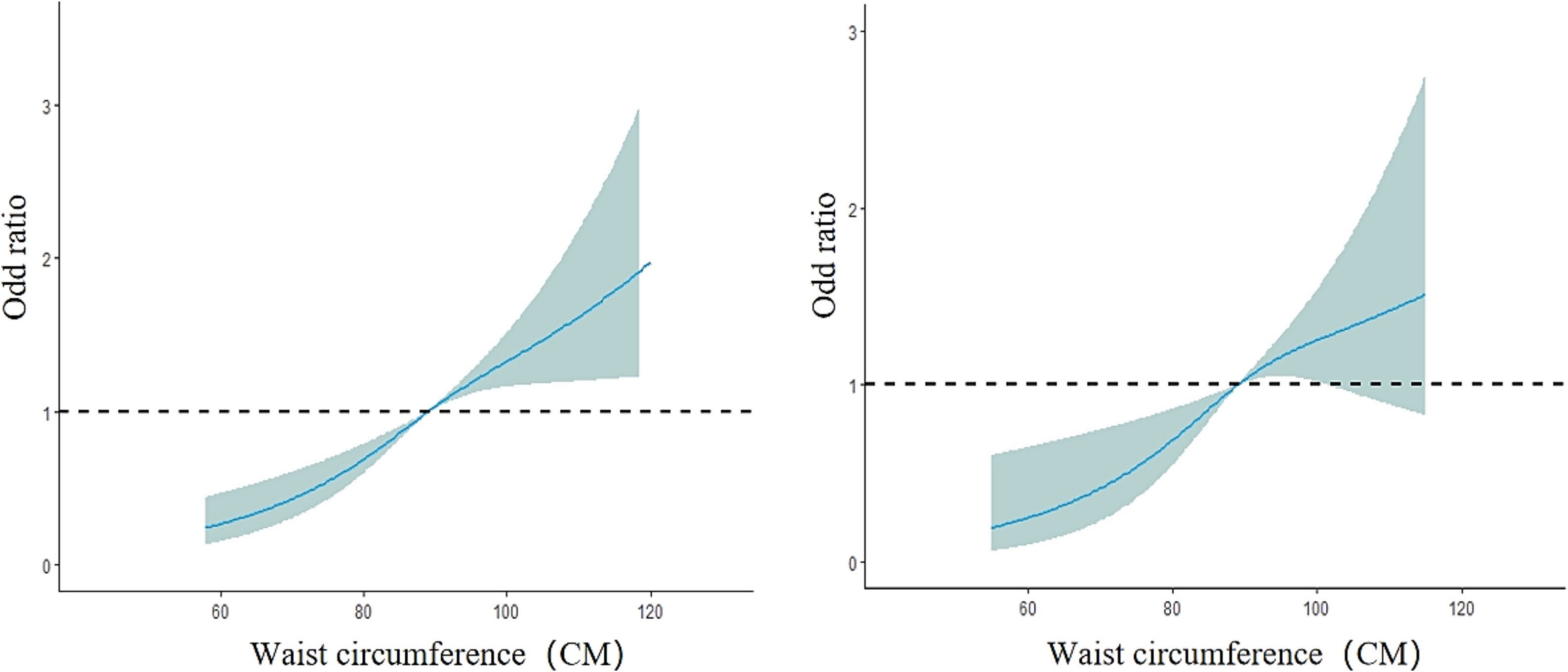
Dose–response relationship between waist circumference and the risk of fatty liver disease in the group under 75 years old (left) and the group 75 years and older (right). The solid blue line represents the adjusted OR values from the restricted cubic spline model, the gray area represents the 95% confidence interval (CI) of the OR values, and the black dashed line represents OR = 1.

The overall model results showed a nonlinear dose–response relationship between waist circumference and the presence of fatty liver disease (nonlinear test, *χ^2^*_overall_ = 6.08, *p* = 0.01). The risk of fatty liver disease in the older adult population increased with increasing waist circumference (*OR* < 1, decreased protective effect). Between 52 and 120 cm of waist circumference, the risk of fatty liver disease gradually increased, but the curve remained relatively flat, as shown in Figure 6. In the subgroup analysis by gender, the male model results showed a nonlinear dose–response relationship between waist circumference and the prevalence of fatty liver disease (nonlinear test, *χ^2^*_male_ = 10.17, *p* < 0.01). The curve change in the male model was similar to that of the overall population model. For males, the risk of fatty liver disease increased more rapidly between 62 and 100 cm of waist circumference and slowed down between 100.2 and 120 cm (*OR* < 1, decreased protective effect). The female model results showed a linear dose–response relationship between waist circumference and the prevalence of fatty liver disease (nonlinear test, *χ^2^*_female_ = 2.82, *p* = 0.09). The risk of fatty liver disease in the female group also increased with increasing waist circumference. Between 54 and 116 cm of waist circumference, the risk of fatty liver disease gradually increased (*OR*<1, decreased protective effect). The curve change in the male model was greater than that in the female model, as shown in Figure 7. In the subgroup analysis by age, both the model for the group under 75 years and the model for the group 75 years and older showed a linear dose–response relationship between waist circumference and the prevalence of fatty liver disease (nonlinear test, *χ^2^*_under 75 years_ = 2.77, *p* = 0.10; *χ^2^*_75 years and older_ = 1.89, *p* = 0.17). The curve change in both models was similar to the overall population model, with the risk of fatty liver disease gradually increasing with waist circumference in both age groups (*OR* < 1, decreased protective effect). The curve change in the model for the group under 75 years was greater than that for the group 75 years and older, as shown in Figure 8.

## 4 Discussion

In this study, the prevalence of fatty liver disease among the older adult was 32.56%, slightly higher than the 30.8% reported for Chinese adults in the study by Liu et al. (21). This may be closely related to the older adult population being the focus of this study, as this demographic typically exhibits a higher risk of developing fatty liver disease (22). Furthermore, Urumqi City’s location in Northwestern China and its distinctive high-fat, high-calorie dietary patterns represent key factors contributing to the elevated prevalence (23). The prevalence of fatty liver disease was higher among older adult females than older adult males (33.40% vs. 31.54%). This may be related to the protective effect of estrogen on fatty liver disease. A decrease in estrogen in postmenopausal women could lead to an increase in the prevalence of fatty liver disease in older adult women (24, 25). The results of this study showed a significant correlation between waist circumference and the risk of fatty liver disease. The results of the multivariate logistic regression analysis indicated that compared with non-central obesity, central obesity significantly increased the risk of fatty liver disease (*OR* = 1.768, 95% *CI*: 1.481–2.112), which is consistent with previous studies (26–28). Current research on fatty liver disease mostly explores the role of general obesity in the etiology of this liver disease, with less discussion on the specific role of central obesity. Some studies suggest that central obesity may pose a greater threat to the development of fatty liver disease than general obesity (29). Waist circumference, as a simple and easily measurable indicator, has been shown to predict the risk of fatty liver disease (30–32). Therefore, using waist circumference and central obesity, defined by waist circumference, as diagnostic indicators to study their association with fatty liver disease can provide more accurate and reliable predictive results, with good potential for public health applications. In this study, random forest and Lasso regression were used to screen variables, and cross validation and correlation analysis were used to remove redundant information. It is conducive to improving the biological significance and statistical robustness of variable selection, effectively controlling collinearity, and enhancing the explanatory power of the model.

The results of the restricted cubic spline model showed a nonlinear dose–response relationship between waist circumference and the risk of fatty liver disease in the older adult population in this study. The risk of fatty liver disease increased with waist circumference (*OR* < 1, decreased protective effect), which is consistent with the results of a cohort study in Korea (33). When the waist circumference was 88.90 cm, the risk of fatty liver disease was 1 (*OR* = 1), and the risk continued to increase slowly when the waist circumference exceeded 88.90 cm. This suggests that maintaining a lower waist circumference in the older adult can help reduce the risk of fatty liver disease. A large cohort study with 16 years of follow-up also indicated that keeping a lower waist circumference is key to preventing fatty liver disease (34). This protective mechanism may relate to metabolic abnormalities associated with increased waist circumference, which signifies visceral adipose tissue accumulation. Such adipose tissue preferentially contributes to insulin resistance, dysregulated lipid metabolism, and chronic inflammation. These processes collectively promote hepatic fat deposition and hepatocyte injury, thereby elevating the risk of developing fatty liver disease (35–37). Additionally, some studies have shown that waist circumference has a mediating effect between certain genotypes and the development of fatty liver disease, suggesting that managing waist circumference can partially control fatty liver disease (33).

The results of the gender-stratified analysis showed that waist circumference in men had a nonlinear dose–response relationship with fatty liver disease, while waist circumference in women had a linear dose–response relationship. In both cases, the risk of fatty liver disease increased with increasing waist circumference. Compared to women, men’s risk of fatty liver disease is more influenced by changes in waist circumference. This may be related to gender differences in fat distribution, as women tend to store fat more in the hips and thighs (38, 39), which may reduce the risk of obesity-related diseases to some extent (40). However, some studies suggest that once diagnosed with fatty liver disease, women are at higher risk of developing advanced fibrosis compared to men (41). In general, men tend to have more unhealthy lifestyle habits than women (42, 43). For example, smoking and alcohol consumption are both risk factors for fatty liver disease. Smoking is significantly associated with fatty liver disease (44, 45), and studies have shown that exposure to second-hand smoke also significantly increases the risk of fatty liver disease (46). The liver, as the main organ responsible for alcohol metabolism, is the primary target of alcohol’s harmful effects (47). Long-term or excessive alcohol consumption significantly increases the fat content in the liver and makes it more prone to liver-related diseases (48–50). These factors undoubtedly lead to a higher risk of fatty liver disease in men with the same waist circumference compared to women. Therefore, among the older adult, both men and women should engage in regular exercise, control their waist circumference, and maintain healthy lifestyle habits to reduce the risk of fatty liver disease.

The age-stratified results showed that both groups exhibited a linear dose–response relationship between waist circumference and the prevalence of fatty liver disease, with increasing waist circumference leading to a higher risk of fatty liver disease. The risk fluctuation of fatty liver disease was greater in the population under 75 years old than in those aged 75 and above. At the same waist circumference, the risk of fatty liver disease was actually lower in older individuals. This is consistent with the findings of Alqahtani et al. (51). Additionally, research shows that the mortality risk from fatty liver disease significantly increases in the 60–74 age group, while the risk does not increase in those over 75 (52). It remains unclear whether the lower prevalence of fatty liver disease in older seniors is due to higher early mortality in those under 75 or due to lifestyle differences across generations.

In summary, waist circumference demonstrates a significant correlation with fatty liver disease risk. Central obesity not only increases fatty liver incidence but is also closely associated with elevated risks of hepatic fibrosis, cirrhosis, and even hepatocellular carcinoma (53, 54). Increased visceral adiposity from waist circumference expansion constitutes a shared risk factor across multiple liver diseases. However, current mainstream management paradigms prioritize weight control and lifestyle/dietary modifications (55). Precise monitoring and maintenance of lower waist circumference levels should therefore be established as core therapeutic objectives and critical efficacy evaluation metrics. This is essential for effectively mitigating disease progression from hepatic steatosis to advanced liver disease. Given its simplicity, strong reproducibility, and patient accessibility for self-monitoring and intervention, waist measurement holds distinct advantages in both clinical practice and public health domains. Compared to complex imaging or biochemical markers, waist circumference—as a robust surrogate for visceral adiposity—offers greater scalability for population-wide screening and longitudinal surveillance. Consequently, integrating waist measurement into routine screening protocols for high-risk groups (e.g., individuals with obesity or metabolic syndrome) and establishing it as a core monitoring indicator in fatty liver disease management will enhance early detection efficacy, optimize intervention strategies, and improve overall management outcomes for fatty liver and related comorbidities.

This study leveraged machine learning-optimized feature selection to investigate the association between waist circumference and fatty liver disease using logistic regression and RCS models, demonstrating substantial clinical and public health significance. However, as a cross-sectional study, it cannot establish causal relationships between waist circumference and fatty liver disease. The sample was exclusively drawn from older adult populations at three medical institutions in Urumqi’s urban area, thereby potentially limiting result generalizability and extrapolation to other populations. Concurrently, fatty liver diagnosis relied solely on ultrasonography without incorporating liver function tests or supplementary imaging modalities, which may have compromised diagnostic precision. Given that this study’s primary objective focused on waist circumference-fatty liver correlations rather than model prediction accuracy, all models employed default parameters without hyperparameter tuning, potentially constraining model performance. Additionally, the evaluation indicators of machine learning models are relatively single. Nominal variables (e.g., smoking/alcohol consumption status) were ordinally encoded in this study, potentially impairing the capture of complex association patterns and introducing result bias. Furthermore, correlation analyses implemented conventional subgroup stratification (sex/age) but omitted deeper stratification (e.g., metabolic phenotypes, ethnicity). Prospective studies should address these constraints to further augment research depth and scientific value.

## Data Availability

The raw data supporting the conclusions of this article will be made available by the authors without undue reservation.
