# Supplementary material for: Association between waist circumference and fatty liver disease in older adult population: a cross-sectional study in Urumqi
Source: Front Public Health. 2025 Jul 8;13:1620261. doi: 10.3389/fpubh.2025.1620261 (PMC12279773; doi:10.3389/fpubh.2025.1620261)
Supplement: Supplementary file 1 [file Table_1.DOCX]

| Item | Classification | Number of People | Fatty Liver Disease | | |
| --- | --- | --- | --- | --- | --- |
|  |  |  | Cases | *χ² Value* | *P Value* |
| Gender | Male | 1766 | 557 | 1.517 | .22 |
|  | Female | 2141 | 715 |  |  |
| Age | Under 75 years | 2702 | 956 | 31.825 | <.001 |
|  | 75 years and older | 1205 | 316 |  |  |
| BMI Classification | Underweight | 58 | 2 | 403.545 | <.001 |
|  | Normal | 1391 | 211 |  |  |
|  | Overweight | 1675 | 634 |  |  |
|  | obesity | 783 | 425 |  |  |
| Central Obesity | Yes | 2321 | 980 | 243.304 | <.001 |
|  | No | 1586 | 292 |  |  |
| Exercise Frequency | Daily | 2136 | 710 | 3.683 | .30 |
|  | More than once a week | 190 | 70 |  |  |
|  | Occasionally | 382 | 122 |  |  |
|  | No Exercise | 1199 | 370 |  |  |
| Hypertension | Yes | 1974 | 707 | 19.295 | <.001 |
|  | No | 1933 | 565 |  |  |
| Diabetes | Yes | 579 | 253 | 38.411 | <.001 |
|  | No | 3328 | 1019 |  |  |
| Dyslipidemia | Yes | 983 | 425 | 68.206 | <.001 |
|  | No | 2924 | 847 |  |  |
| Smoking Status | Never smoked | 3480 | 1107 | 8.547 | .01 |
|  | Quit smoking | 111 | 40 |  |  |
|  | Smoking | 316 | 125 |  |  |
| Drinking Frequency | Never | 3568 | 1140 | 8.959 | .03 |
|  | Occasionally | 233 | 85 |  |  |
|  | Frequently | 51 | 23 |  |  |
|  | Daily | 55 | 24 |  |  |
| Dietary Habits | Balanced diet | 3779 | 1240 | 3.635 | .30 |
|  | Meat-based | 44 | 12 |  |  |
|  | Vegetarian-based | 79 | 19 |  |  |
|  | Salt-lover | 5 | 1 |  |  |
| ECG | Normal | 2292 | 638 | 56.282 | <.001 |
|  | Abnormal | 1615 | 634 |  |  |
| Aortic Sclerosis | Yes | 719 | 284 | 19.341 | <.001 |
|  | No | 3188 | 988 |  |  |

**Table 1. Prevalence of Fatty Liver Disease in Elderly Individuals with Different Characteristics**
